# Bamboo nodes show nature's wisdom

**DOI:** 10.1093/nsr/nwac270

**Published:** 2022-11-28

**Authors:** Helmut Cölfen

**Affiliations:** Department of Chemistry, University of Konstanz, Germany

Bamboo (*Phyllostachys edulis*) is a biological structural material featuring tall stalks and excellent mechanical properties [1]. It has been applied to many engineering areas such as construction, transportation and energy [2]. Laminates are the main material processing and application forms, where the tubular bamboo internodes with unidirectionally arranged fibrous vascular bundles play key roles [1,2]. Under the huge internode demand for processing structural materials, short bamboo nodes that segment and block the tubular internodes are often discarded or broken directly to connect all internodes. Are the bamboo nodes really of no significance or use?

**Figure 1.**
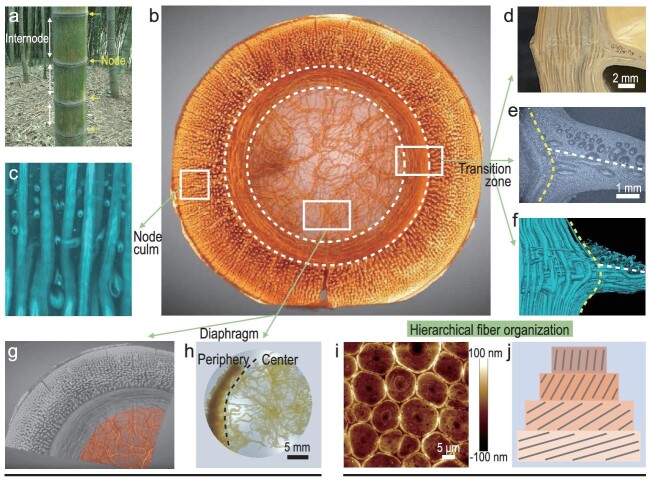
Fiber-based structure of bamboo node. (a) Digital image of bamboos, highlighting short nodes and tall internodes. (b) Micro-CT image of one bamboo node, showing a complicated fiber-based structure. (c) Micro-CT image of one node culm. (d–f) Digital, optical and micro-CT images of the transition zone between node culm and diaphragm. (g and h) Micro-CT and digital images of the central diaphragm. (i) AFM image of one single vascular bundle, showing densely packed microfibers. (j) Schematic illustration of one single microfiber, showing twist-aligned nanofibers. Reprinted from Ref. [3].

Recently, a team led by Prof. Shu-Hong Yu at the University of Science and Technology of China published an excellent and solid work on scientifically understanding the microstructure of the bamboo node and exploring its potential uses (Fig. 1a) [3]. Through the combination of multi-scale imaging techniques including optical microscopy, X-ray CT microscopy, SEM and atomic force microscopy (AFM), they clearly showed the complex fibrous vascular bundle-based structure of the bamboo node (Fig. 1b), which is a manifestation of the ingenious structural design of the bamboo node. Based on the determined structures and specific mechanical tests and simulations, they proposed three kinds of fiber-reinforced structural schemes: spatially interlocked structures at the node culm (Fig. 1b)and c), a triaxial interconnected scaffold structure at the transition zone between node culm and diaphragm (Fig. 1b)and d–f) and isotropic interwoven structures at the central diaphragm (Fig. 1b, g and h). The interlocking structure at the node culm, coupled with the familiar gradient structure along the radial direction, can directly resist external loads; the scaffold structure at the transition zone can facilitate stress transfer and act as a bridge to maintain internal and external stability; the isotropic interwoven structure at the soft diaphragm can facilitate the dissipation of external transferable energy. These structures are position-dependent (Fig. 1b), reflecting the organism's ability to spatially allocate limited resources for optimal structural stability. In general, like abalone shells [4], teeth [5], fish scales [6] and crustacean exo/endocuticles [7], the bamboo node also conforms to the general structural design principle of biological materials, which is rigid/hard on the outside and tough/soft on the inside.

It is worth noting that fibrous vascular bundles of hundreds of microns in diameter are hierarchically assembled from cellulose molecules, nanofibers and microfibers (Fig. 1i)and j). The structural hierarchy of fibrous vascular bundles further enriches the connotation of the proposed structural reinforcement schemes. These multi-scale reinforcement schemes are exactly what is needed for engineering structural materials now [1,8]. Interestingly, besides the characteristic fibrous hierarchy, vascular bundles also have multi-scale channels for water and nutrient transport. Using the structure–function integration of vascular bundles within bamboo nodes, Yu's team skillfully fabricated a kind of node-based photo-thermal water-evaporation device, which is structurally stable, shows high principles of performance and is expected to alleviate the shortage of fresh water.

In summary, this timely research article by Yu *et al.* reveals optimized hierarchical fiber arrangements of the bamboo node demonstrating the wise construction principles of nature. This has great implications for heterogeneous fiber-reinforced structural design, high-value biomass conversion and sustainable development.
